# Comparison of leak fraction between the laryngeal mask airway and endotracheal tube during anesthesia: a single-center retrospective study

**DOI:** 10.1007/s00540-024-03364-y

**Published:** 2024-06-22

**Authors:** Seiichi Azuma, Masaaki Asamoto, Shinichi Akabane, Mariko Ezaka, Mikiya Otsuji, Kanji Uchida

**Affiliations:** 1 Department of Anesthesiology and Pain Relief Center, The University of Tokyo Hospital, Hongo 7-3-1, Bunkyo-Ku, Tokyo, 113-8655 Japan; 2 Graduate School of Medicine, The University of Tokyo, Tokyo, Japan; 3 Department of Anesthesiology, Tokyo Teishin Hospital, Tokyo, Japan

**Keywords:** Laryngeal mask, Leakage, Positive pressure ventilation, Tracheal intubation

## Abstract

The use of the laryngeal mask airway (LMA), which offers the benefits of ease in insertion and prevention of tracheal damage, is associated with a risk of flow leakage. This study analyzed our extensive database to compare leakage associated with the use of LMA and endotracheal tube (ETT). Adult patients who underwent chest wall, abdominal wall, inguinal region, limb, transurethral, or transvaginal surgery and received either LMA or ETT between January 2007 and March 2020 were included. The leak fraction was calculated as (inspiratory tidal volume−expiratory tidal volume)/(inspiratory tidal volume) × 100% every minute during intraoperative stable positive pressure ventilation. The median leak fraction was calculated for each case. The leak fraction in the LMA group demonstrated a left-skewed distribution with a larger proportion of excessive leak fraction. The leak fraction in the LMA group (median, 7.9%; interquartile range, 4.8–11.4%) was significantly lower than that in the ETT group (median, 9.1%; interquartile range: 5.5–12.4%; *P* < 0.001). This tendency was consistent across subgroups divided by sex, age, type of surgery, and ventilation mode. We propose that LMA provides leakage comparable to or less than ETT in most cases if stable positive pressure ventilation is achieved.

The laryngeal mask airway (LMA), extensively used in general anesthesia, provides advantages, such as quicker and easier insertion, reduced risk of tracheal damage, and smoother emergence over endotracheal tube (ETT) intubation [1, 2]. On the other hand, LMA may not consistently provide an ideal fit [3–5], raising concerns regarding the higher risk of flow leakage [6, 7]. This poses the risk of microbial transmission and environmental contamination of anesthetic gasses, exposing healthcare professionals to potential harm. Previous studies have shown that LMA can be safely used with low-flow anesthesia with similar leakage to ETT [8, 9]. As these reports were limited to specific situations with a relatively limited population, this retrospective observational study aimed to compare leakage associated with the use of LMA and ETT in a large-scale clinical practice using our extensive data.

This study was conducted as part of the comprehensive research project “Examining Optimal Physiological Parameters for Ideal Perioperative Management Utilizing Medical Records in the Department of Anesthesiology and Pain Relief Center,” approved by the Research Ethical Committee of the Faculty of Medicine at the University of Tokyo (2023166NI) and registered in the Japan Registry of Clinical Trials (jRCT1030230547). We used an opt-out enrollment method through the institutional website to obtain informed consent. We included data from patients aged ≥ 18 years who underwent surgical procedures in which both LMA and ETT were commonly applied. These included breast, gastrointestinal, gynecological, plastic, urological, and orthopedic surgeries involving the chest wall, abdominal wall, inguinal region, limbs, or abdominal viscera with transurethral or transvaginal approaches, which were performed in the supine or lithotomy position. We included cases managed using the Fabius Tiro™ (Dräger, Lübeck, Germany) anesthesia machine at the University of Tokyo Hospital between January 2007 and March 2020, for which the ventilation circuit settings were standardized. The choice of the airway device was left to the attending anesthesiologist. We focused on cases managed using LMA (LMA group) and normal ETT (ETT group). Cases in which LMA was switched to ETT or vice versa were excluded. The LMA devices used in this study were LMA Classic™ (Teleflex Medical, Dublin, Ireland), LMA ProSeal™ (Teleflex Medical, Dublin, Ireland), LMA Supreme™ (Teleflex Medical, Dublin, Ireland), and i-gel™ (Intersurgical, Wokingham, UK). The ETT used was the Portex™ series (Smiths Medical, Minneapolis, MN, USA).

Minute-by-minute instantaneous data of inspiratory and expiratory tidal volumes were obtained using a flow-volume sensor (GF-220™, Nihon Kohden, Japan), and gas sampling at 200 mL/min was automatically corrected. We used only data recorded during surgery, to avoid the period around intubation and extubation. The leak fraction was calculated as

$$\frac{{\text{(inspiratory tidal volume)} - \text{(expiratory tidal volume)}}}{\text{(inspiratory tidal volume)}}\, \times \,100\,\left( \% \right)$$

for all data points satisfying the stable positive pressure ventilation criteria, defined as respiratory rate, 5–20 breaths/min; inspiratory tidal volume, 200–800 mL; peak pressure, 5–30 cmH_2_O; minimum pressure, ≥ 0 cmH_2_O; and end-tidal carbon dioxide, 30–50 mmHg. Cases without ≥ 10 data points satisfying the criteria were excluded. The median value was obtained for each case and compared between the LMA and ETT groups.

Because the leak fraction was not expected to be normally distributed, we calculated the median value and interquartile range within each group and compared them using the Mann–Whitney *U* test. We compared the leak fraction between subgroups based on age, sex, type of surgery, and ventilation mode to explore whether these factors altered the results. Age was divided by the median of the entire dataset. The distribution of these factors between the groups was examined using the chi-square test. Analyses were conducted using SciPy version 1.11.4 for Python (version 3.11.6; Python Software Foundation, Wilmington, DE, USA). A two-sided *P* value of < 0.05 indicated statistical significance.

Overall, 10,907 cases (LMA group: 3402, ETT group: 7505) were included, excluding 35 that involved switching from LMA to ETT or vice versa. Among them, 1250 cases (LMA group: 356, ETT group: 894) were excluded because < 10 data points were recorded. Additionally, 795 cases (LMA group: 664, ETT group: 131) without ≥ 10 data points satisfying the stable positive pressure ventilation criteria were excluded. Finally, eight cases (LMA group: four, ETT group: four) exhibiting an extreme leak fraction of < − 50% or > 100% were excluded. Accordingly, 8854 cases (LMA group: 2378, ETT group: 6476) were analyzed. The LMA group comprised 1761 females and 617 males, with a mean ± standard deviation age of 52.8 ± 16.9 years. The ETT group comprised 4700 females and 1776 males, with a mean ± standard deviation age of 60.1 ± 15.5 years. Sex distribution did not differ significantly between the groups (*P* = 0.17), with more females in both groups, whereas age was significantly higher in the ETT group than in the LMA group (*P* < 0.001). We identified 829 cases using volume control ventilation (VCV) and 824 using pressure control ventilation (PCV) with LMA, whereas 4119 used VCV and 969 used PCV with ETT, excluding cases with both or neither modes recorded. VCV was more frequent in the ETT group than in the LMA group (*P* < 0.001).

The leak fraction was more left-skewed in the LMA group than in the ETT group (Fig. 1). The leak fraction was significantly lower in the LMA group (median: 7.9%, interquartile range: 4.8–11.4%) than in the ETT group (median: 9.1%, interquartile range: 5.5–12.4%; *P* < 0.001). Subgroup analyses demonstrated a consistent tendency for less leakage with LMA than with ETT (Table 1). The magnitude of the differences varied among subgroups. Differences were significant for the female, ≥ 60 years, gynecological, orthopedic, and VCV subgroups but not for the other subgroups.

**Fig. 1:**
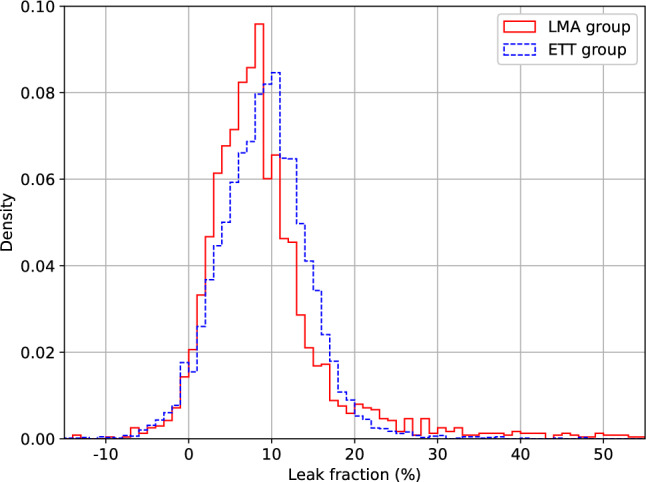
Distribution of leak fraction. The density values represent the relative frequency per each 1% leak fraction interval, with the total area under the curve equaling one. Red solid line, LMA group; blue dashed line, ETT group; *LMA* laryngeal mask airway, *ETT* endotracheal tube

**Table 1:** Leak fraction (%) with laryngeal mask airway (LMA) or endotracheal tube (ETT) among subgroups divided by sex, age, type of surgery, and ventilation mode

	LMA group	ETT group	*P* value
Sex			
Female	8.2 (5.0–11.6) (*n* = 1761)	9.8 (6.4–12.8) (*n* = 4700)	< 0.001
Male	7.0 (3.9–10.6) (n = 617)	7.1 (3.8–10.6) (*n* = 1776)	0.71
Age			
< 60 years	7.9 (4.7–11.1) (*n* = 1490)	8.3 (4.9–11.4) (*n* = 2832)	0.50
≥ 60 years	8.0 (4.8–11.7) (*n* = 888)	9.8 (6.3–12.9) (*n* = 3644)	< 0.001
Type of surgery			
Breast	7.0 (4.4–10.8) (*n* = 244)	8.3 (5.1–11.2) (*n* = 1297)	0.071
Gastrointestinal	7.4 (4.8–11.1) (*n* = 64)	7.9 (3.9–11.3) (*n* = 748)	0.80
Gynecological	8.2 (5.0–11.4) (*n* = 1025)	9.4 (5.6–12.8) (*n* = 353)	0.014
Plastic	7.8 (5.1–13.6) (*n* = 86)	8.7 (5.2–12.2) (*n* = 799)	0.68
Urological	7.2 (4.3–10.9) (*n* = 598)	8.3 (4.1–11.4) (*n* = 564)	0.38
Orthopedic	8.4 (5.2–12.4) (*n* = 361)	10.1 (6.7–13.2) (*n* = 2715)	< 0.001
Ventilation mode			
Volume control	8.7 (5.9–12.1) (*n* = 829)	9.7 (6.3–10.9) (*n* = 4119)	< 0.001
Pressure control	7.5 (4.3–10.9) (*n* = 824)	7.8 (4.4–11.3) (*n* = 969)	0.86

Data are shown as the median (interquartile range) (n = number of cases)

This single-center retrospective study showed significantly less leakage with LMA than with ETT, reinforced by the consistent trend across subgroups. We acknowledge that the small difference, likely overestimated by the higher age and more frequent choice of VCV with ETT than with LMA, has limited clinical significance. However, our research contributes by extending previous results of similar leakage between LMA and ETT [8, 9] from controlled to pragmatic settings and by providing comparisons across various subgroups.

The varying influences of demographic factors between groups can be attributed to the different regions associated with leakage: the larynx for LMA and lower trachea for ETT. The shape of the trachea and the configuration of the tracheal cartilage differ between males and females [10, 11], and aging increases the area and distorts the roundness of the trachea [10, 12]. Such anatomical differences likely contributed to the differences in leakage among subgroups within the ETT group. Although sex and age also cause anatomical differences in the larynx [13, 14], their influence on leakage was less prominent with LMA than with ETT. Consequently, female or older patients exhibited more leakage with ETT and relatively unchanged leakage with LMA, resulting in a more significant difference between LMA and ETT in these subgroups than in male or younger patients. The significantly larger leakage in the gynecological subgroup than in the other subgroups in the ETT group is likely attributable to the sex distribution, whereas that in the orthopedic subgroup may be attributable to the vibration associated with surgery. The larger leakage with VCV than with PCV agrees with the study using facemasks [15], which may be attributed to higher peak pressure. The smaller difference between the groups with PCV and those with VCV in the LMA group than the ETT group may be due to the preference for PCV in cases with large leakage with LMA.

We note important considerations for applying our results regarding excessive leakage with LMA, which may occur due to poor fit [3–5] or vocal cord closure [16]. First, our data with pragmatic settings essentially included individualized efforts to adjust ventilation setting, cuff pressure, and anesthetic agents including additional muscle relaxants. More efforts may have been devoted to cases with LMA. Second, more patients were excluded from the LMA group (664/3046) than from the ETT group (131/6611) due to < 10 data points satisfying the stable positive pressure ventilation criteria. Third, taking the median value within cases ignored temporary excessive leakage. Finally, our comparison based on median values ignored the higher proportion of large leakage with LMA, as indicated by the left-skewed distribution. Therefore, we emphasize that our results only apply to situations without excessive leakage and that when choosing LMA, potential efforts in managing excessive leakage, including switching to ETT, must be considered.

This study has some limitations. The results in pragmatic settings depend on patient characteristics and institutional practices, some of which we have described, while the lack of other information limits the interpretation of our findings. Notably, since cuff pressure was not routinely recorded, the cuff pressure of ETT may have been relatively low, potentially leading to increased leakage. We did not assess the rate of excessive leakage occurrence or the individualized efforts for managing leakage because of our limited retrospective records. The potential tendency to choose ETT for patients with severe complications or those managed by junior residents may have caused a bias. The flow-volume sensor may cause different biases depending on different flow patterns between VCV and PCV or different resistances between LMA and ETT. We did not perform multivariable analyses, which was challenging because of the non-normal distribution of the leak fraction and imbalanced distributions of demographic factors.

In summary, our extensive data revealed less leakage with LMA than with ETT in pragmatic settings. This tendency was consistent across subgroups and was more evident in female or older subgroups. We propose that the risk of flow leakage with LMA is primarily attributed to excessive leakage in specific situations, whereas in most cases where stable positive pressure ventilation is achieved, LMA provides comparable or less leakage than ETT.

## Data Availability

The data are available from the corresponding author upon reasonable request.
